# Application of Multispectral Imaging to Determine Quality Attributes and Ripeness Stage in Strawberry Fruit

**DOI:** 10.1371/journal.pone.0087818

**Published:** 2014-02-04

**Authors:** Changhong Liu, Wei Liu, Xuzhong Lu, Fei Ma, Wei Chen, Jianbo Yang, Lei Zheng

**Affiliations:** 1 School of Biotechnology and Food Engineering, Hefei University of Technology, Hefei, China; 2 Intelligent Control and Compute Vision Lab, Hefei University, Hefei, China; 3 Institute of Rice Research, Anhui Academy of Agricultural Sciences, Hefei, China; 4 School of Medical Engineering, Hefei University of Technology, Hefei, China; ISA, Portugal

## Abstract

Multispectral imaging with 19 wavelengths in the range of 405–970 nm has been evaluated for nondestructive determination of firmness, total soluble solids (TSS) content and ripeness stage in strawberry fruit. Several analysis approaches, including partial least squares (PLS), support vector machine (SVM) and back propagation neural network (BPNN), were applied to develop theoretical models for predicting the firmness and TSS of intact strawberry fruit. Compared with PLS and SVM, BPNN considerably improved the performance of multispectral imaging for predicting firmness and total soluble solids content with the correlation coefficient (*r*) of 0.94 and 0.83, SEP of 0.375 and 0.573, and bias of 0.035 and 0.056, respectively. Subsequently, the ability of multispectral imaging technology to classify fruit based on ripeness stage was tested using SVM and principal component analysis-back propagation neural network (PCA-BPNN) models. The higher classification accuracy of 100% was achieved using SVM model. Moreover, the results of all these models demonstrated that the VIS parts of the spectra were the main contributor to the determination of firmness, TSS content estimation and classification of ripeness stage in strawberry fruit. These results suggest that multispectral imaging, together with suitable analysis model, is a promising technology for rapid estimation of quality attributes and classification of ripeness stage in strawberry fruit.

## Introduction

Strawberry fruit (*Fragaria*×*ananassa* Duch.) is an economically important fruit which is more popularly consumed fresh, as well as used for garnishing cakes and pastries, flavored for juices and milk products, and processed into jams and other products. Therefore, together with the recent attention for food quality and safety, technologies for estimating the fresh quality of strawberry fruit are being sought [1]. At present, fruit are sorted manually or automatically on the basis of their external quality features. However, internal quality attributes such as firmness, sweetness, acidity and flavor are very important in the quality evaluation industries. In addition, since the strawberry is a non-climacteric fruit, in order to achieve good quality, it is essential to harvest at the optimum stage of ripening [2]. Currently, many objective criteria for judging maturity of strawberry have been used, for example, firmness, total soluble solids, titrable acidity, and determination of total anthocyanins. However, standard methods for these quality measurements are mostly destructive, slow, and prone to operational error. In order to overcome these disadvantages, nondestructive methods, especially those based on optical properties, are urgently required.

Near infrared spectroscopy (NIRS) is a nondestructive technique and highly suited to the measurement of quality attributes in fresh fruits and vegetables. It is a chemical-free, rapid measuring method with limited sample preparation, and enables the simultaneous determination of several attributes [3], [4]. Recently, many published studies address the application of NIRS technology to determine firmness, soluble solids content, titratable acidity, pH and soluble sugar components in strawberry fruit [4]–[7]. However, NIR spectrometers only detect a small portion of the fruit; therefore, the spectra are sometimes not representative for the whole fruit.

Hyperspectral imaging is an emerging nondestructive technology that integrates conventional imaging and spectroscopy to attain both spatial and spectral information from an object simultaneously [8], [9]. In strawberry fruit, Nagata et al. [1] had developed prediction models for firmness and soluble solids content using hyperspectral imaging in the visible range (450–650 nm). Similarly, Tallada et al. [10] conducted a hyperspectral imaging investigation for firmness in strawberry fruit using NIR hyperspectral imaging. Recently, ElMasry et al. [11] determined moisture content, total soluble solids content and pH in strawberry fruit using hyperspectral imaging in the visible and near-infrared region. However, the rich information in hyperspectral imaging results in difficulties in data processing, which makes it hard for industrial online applications. To overcome this problem, a simplified version called multispectral imaging (MSI) is available. This technology has recently been applied as a powerful process analytical tool for rapid, nondestructive inspection of internal and external attributes in various fruits and vegetables [12]–[20]. However, to our knowledge, there is no published data on the multispectral imaging for determination of quality attributes and ripeness stage in strawberry fruit. Furthermore, all of above predictions of quality attributes in strawberry fruit based on spectral imaging technique have been made using PLS analysis or MLR analysis. New regression methods such as support vector machine (SVM) and back propagation neural network (BPNN) appear promising in that they enable the non-linearity of data to be modeled using local or specific equations which could improve prediction models. Therefore, the main objective of this study was to assess the application of multispectral imaging for predicting the major quality attributes and ripeness stage in strawberry fruit, and comparing the performance of prediction models obtained using PLS, SVM and BPNN.

## Materials and Methods

### Sample Preparation

Unripe (white color) and ripe (orange-red color) strawberry fruit (*Fragaria × ananassa* Duch.) were harvested manually from local commercial greenhouse in Hefei City, China in March 2013. The study was carried out on private land and the owner of the land gave permission to conduct the study on this site. Furthermore, the field studies did not involve endangered or protected species. Two hundred and ten fruit (including seventy unripe fruit and one hundred and forty ripe fruit) with uniform shape and size and free from any abnormal features such as defects, diseases, and contaminations were selected, and transported for a short distance to the laboratory. Unripe and seventy ripe fruit were then acquired multispectral images immediately. In order to generate overripe (dark red color) fruit, seventy ripe fruit were kept in room temperature for 2 days. All green calyxes were completely removed from the tested fruit.

A penetration test was performed on the skin of whole fruit using a TA.XT2i texture analyzer (Stable Micro Systems, Guildford, UK) with a 6 mm diameter cylindrical probe. Samples were penetrated to a depth of 7 mm. The speed of the probe was 1.0 mm/s during the pretest as well as during penetration. From the force versus time curves, firmness was defined as the maximum force in newtons (N). Each fruit was measured at two perpendicular sides. Total soluble solids content of strawberry fruit was measured at room temperature using a hand held refractometer (WYT-32; QuanzhouOptical Co. Ltd, Quanzhou, China). A drop of clear juice was placed on an absolutely dry and clean refractometer prism, and a reading was taken directly. The total soluble solids content was expressed as a percentage on the Brix scale. Each fruit was measured in triplicate and both of these measurements were performed immediately after multispectral imaging measurements.

### Multispectral Imaging System

**Figure 1:**
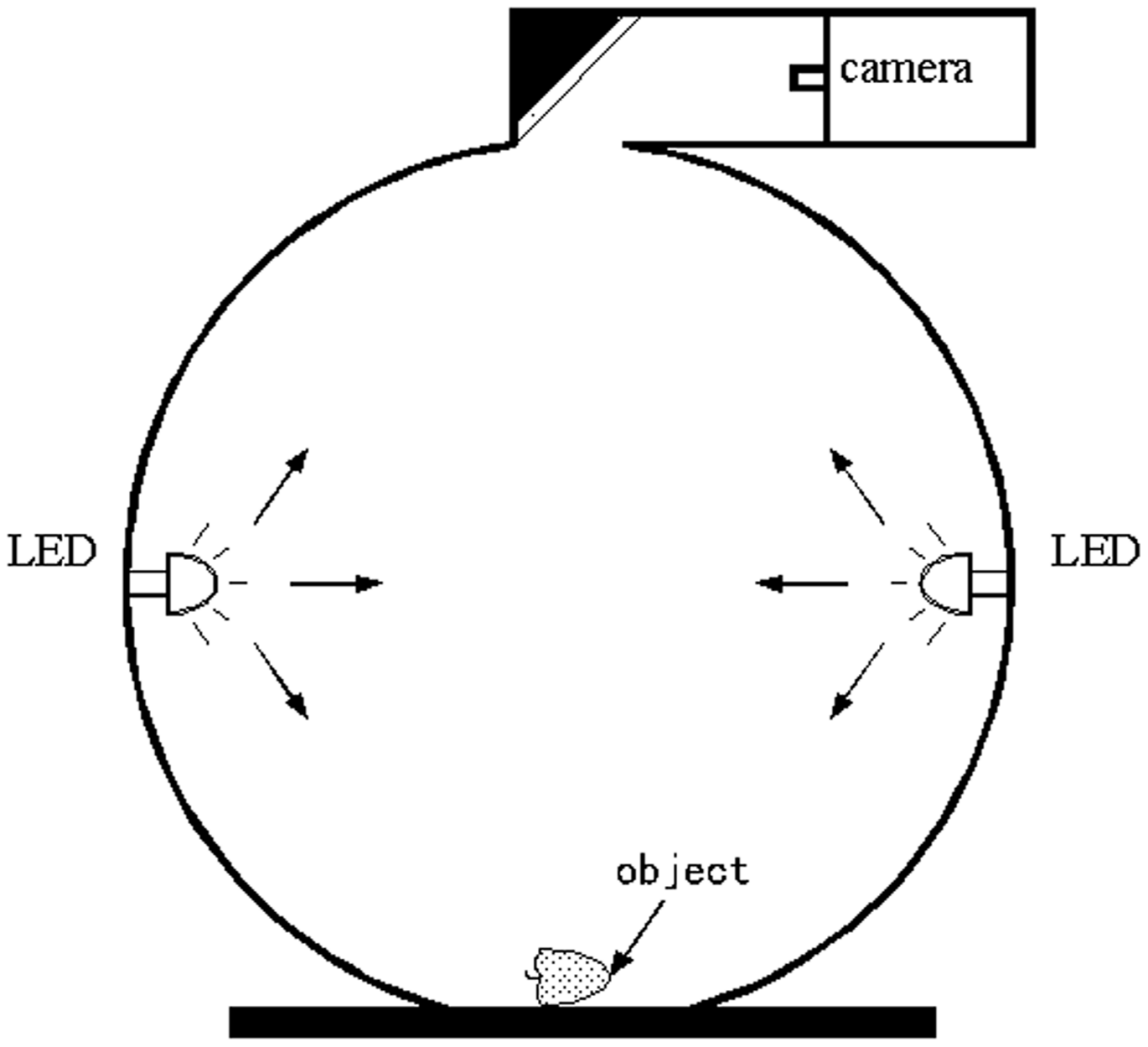
Principal setup of the multispectral imaging system. An integrating sphere with a matte white coating ensures optimal lighting conditions. The light emitting diodes located in the rim of the sphere ensures narrowband illumination. The image acquisition is performed by a monochrome grayscale CCD camera mounted in the top of the sphere.

The data acquisition was done using VideometerLab equipment (Videometer A/S, Hørsholm, Denmark) which acquires multispectral images at 19 different wavelengths from the visual (VIS) region to the lower wavelengths of the NIR region and the detailed information of the measured wavelength were 405, 435, 450, 470, 505, 525, 570, 590, 630, 645, 660, 700, 780, 850, 870, 890, 910, 940 and 970 nm. The majority of the wavelengths are in the visual region. Fig. 1 shows the principal setup of the system. The acquisition system records surface reflections with a standard monochrome charge coupled device chip, nested in a Point Grey Scorpion camera. The object of interest is placed inside an integrating sphere with a matte white coating to ensure that the light is scattered evenly with a uniform, diffuse light at illumination. At the rim of the sphere light emitting diodes (LEDs) are positioned in the pattern of side by side distributing the LEDs at the specific wavelength uniformly around the entire rim. The LEDs are strobing successively, resulting in an image for each LED of dimensionality 1280×960. The system is first calibrated radiometrically using both a diffuse white and dark target followed by a light setup based on the type of object to be recorded. The system is geometrically calibrated with a geometric target to ensure pixel correspondence for all spectral bands [21]–[23]. Each multispectral image consists of 19 separate images, which are recorded at 19 different wavelengths. Segmenting images into distinct regions is an important preprocessing step in image analysis. Image segmentation was performed using the VideometerLab software version 2.12.23. To remove the image background, all items except the strawberry fruit were removed by a Canonical Discriminant Analysis (CDA) [24] and segmented using a simple threshold. The decomposed result with desired features highlighted was then segmented easily using an adaptive thresholding technique known as Otsu adaptive thresholding method [25]. The images of strawberry fruit samples without the background could be transformed to spectra based on a mean calculation. Thus each image contributed with a single spectrum for the model calibration.

### Data Analysis

#### Spectral analysis for predicting quality attributes

To develop a model between the extracted spectra and reference quality attributes (firmness and total soluble solids content), partial least square (PLS), support vector machine (SVM) and back propagation neural network (BPNN) were applied to build the model of prediction. PLS is a linear regression method for multivariate calibration. It has been widely applied in fruits and vegetables analysis and obtained favorable results. Leave-one-out cross-validation was used to evaluate the quality and to prevent over-fitting of calibration models. The optimal number of latent factors was determined at the lowest value of predicted residual error sum of squares. Latent factors can eliminate noises and random errors in the original data and account as much as possible for the variability of the original variables. The theory of PLS has been presented extensively in [26].

Support vector machine (SVM) proposed by Cortes and Vapnik [27] is a learning algorithm used for classification and regression tasks. It has been effectively used to perform multivariate function estimation, non-linear classification, or non-linear regression. SVM model is a representation of the calibration sample set as vectors in space mapped so that the samples from the separate categories are divided by a clear gap that is as wide as possible. New samples from cross-validation or a test set are then mapped into that same space. Based on which side of the gap between classes they fall, they are predicted to belong to one category or another. SVM show high performance in practical applications when solving sophisticated classification problems. In SVM regression, the prediction errors are penalized linearly with the exception of a deviation below a certain value, ε, according to Vapnik’s ε-insensitive loss function. It is possible to use kernel functions, or kernels, that enable non-linear regression in a very efficient way. The values of ε and the parameter *C* (regularization parameter) have to be defined by the user; both are problem- and data-dependent. Cross-validation by leave-one-out procedure was performed during the validation step in order to define the optimum number of parameters that should be kept in the model and to detect any outliers. Compared with other methods, SVM does not require a large number of training samples for model development and is not affected by the presence of outlier [28]. The theory of SVM has been described extensively in Sun et al. [29] and Devos et al. [30]. But the major drawbacks of SVM are that training the model requires optimization of the regularization and kernel meta-parameters in order to control the risk of overfitting and the complexity of the boundary [30].

Back propagation neural network (BPNN) is a type of nonlinear neural network that can solve complex problems more accurately than linear techniques. However, BPNN suffers from difficulties with generalization because of overfitting. In addition, it needs too much time and efforts to determine control parameters including hidden nodes, iteration times, etc. [31]. Principal component analysis-back propagation neural network (PCA-BPNN) model could avoid some of these disadvantages. PCA was performed firstly to extract information from the whole spectral regions, and the few principal components were used to be the BPNN input layer. Several network architectures were tested by varying the number of neurons in the hidden layer with different initial weights. The output of network expresses the resemblance that an object corresponds with a training pattern. The optimal parameters of the hidden nodes, the goal error and iteration times were determined by the least prediction error. The theory of BPNN has been described in detail in Dubey et al. [32].

A total of 210 fruit were included in the analysis; 162 fruit including 54 unripe samples, 54 ripe samples and 54 overripe samples were used as a calibration set for developing models, and 48 fruit including 16 unripe samples, 16 ripe samples and 16 overripe samples were used for model validation to verify the prediction power of the calibration models. Models were calibrated using leave-one-out cross-validation (LOOCV). The quality of the calibration model was evaluated by the standard error of calibration (SEC), standard error of prediction (SEP) and the correlation coefficient (r) between the predicted and measured value of the attribute. A good model should have a high correlation coefficient (r), and a low SEC and SEP with little difference between them. These criteria are defined as follows:
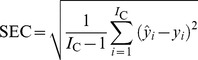
(1)

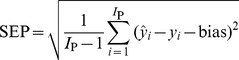
(2)

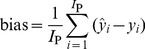
(3)Where, 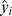 is the predicted value of an attribute in fruit number i; *y_i_* is the measured value of an attribute in fruit number i; *I*_C_ is the number of fruit (spectra) in the calibration set; and *I*_P_ is the number of fruit (spectra) in the validation set.

#### Spectral analysis for classifying ripeness stage

SVM and principal component analysis-back propagation neural network (PCA-BPNN) were applied to classify fruit ripeness stage. For the PCA-BPNN model, PCA was performed firstly to extract information from the whole spectral regions, and the few principal components were used to be the BPNN input layer. A total of 210 fruit were included in the analysis. Of these, 150 fruit including 50 unripe samples, 50 ripe samples and 50 overripe samples were used for the development of the classification models, and 60 fruit including 20 unripe samples, 20 ripe samples and 20 overripe samples were used for model validation to verify the prediction power of the calibration models.

### Statistical Analysis

All image analyses and statistics were carried out using Matlab 2009 (The Mathworks Inc., Natick, MA, USA) and Origin 8.5.

## Results and Discussions

### Reflectance Spectral Analysis

**Figure 2:**
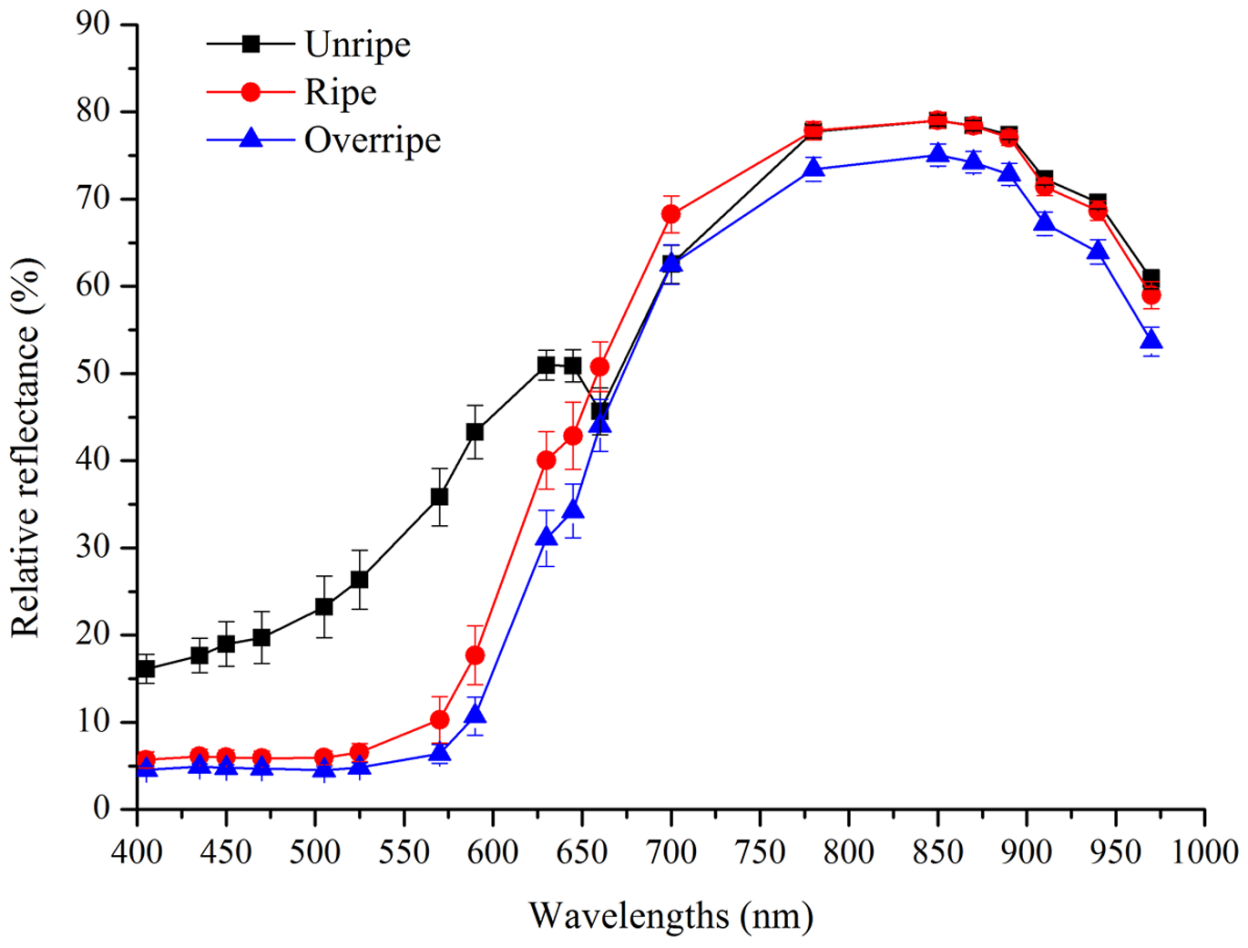
Average reflectance from the multispectral images of unripe, ripe and overripe strawberry fruit. *Vertical bars* represent standard deviations from seventy measurements.

Fig. 2 shows the average relative reflectance spectra in a range of 405–970 nm of strawberry fruit sampled at three ripeness stages. In despite of ripeness stage, the reflectance curves of strawberry fruit were rather smooth across the entire spectral region. Anthocyanins and chlorophyll which represent the color characteristics in the strawberry fruit have previously been identified at around 520 and 680 nm wavelength, respectively [33], [34]. ElMasry et al. [11] reported that NIR absorption at around 840 and 960 nm were related to sugars and water content, respectively. In the present study, the chlorophyll absorption band at 660 nm was almost absent in ripe and overripe fruit due to the chlorophyll degradation in this fruit. Meanwhile, the relative reflectance at anthocyanin absorption band (at 525 nm) was much lower in ripe and overripe fruit than those in unripe fruit, indicating that the anthocyanin content in the ripe and overripe fruit are much higher. The results were similar to those reported by ElMasry et al. [11], who showed that the anthocyanins in the ripe and overripe fruit were higher than unripe fruit. Previous research identified that the main contributors to total soluble solids content were sugars and the sugar content accounted for 57% to 82% of total soluble solids content [5]. The relative reflectance at sugars absorption band (at 850 nm) was much lower in overripe fruit than those in unripe and ripe fruit, which may be attribute to the higher total soluble solids content in overripe fruit (8.63°Bx) than those in unripe and ripe fruit (7.03 and 7.79°Bx, respectively).

### Quality Attributes Prediction

**Figure 3:**
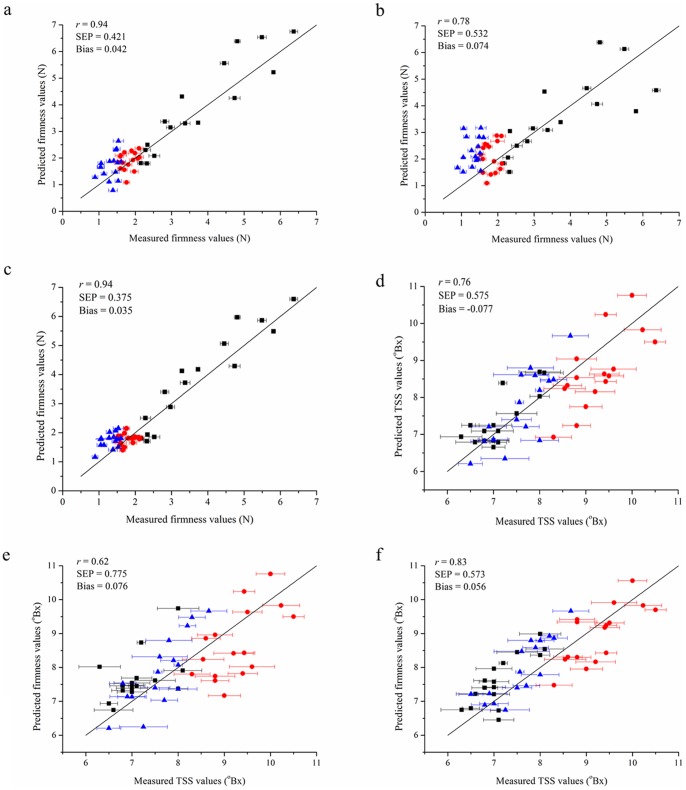
Comparison of predicted and measured values for the prediction sets. (a) prediction of firmness using PLS model; (b) prediction of firmness using SVM model; (c) prediction of firmness using BPNN model; (d) prediction of TSS using PLS model; (e) prediction of TSS using SVM model; (f) prediction of TSS using BPNN model. The black square indicated the unripe strawberry fruit; the red circle indicated the ripe strawberry fruit; the blue triangle indicated the overripe strawberry fruit.

Table 1 shows values for range, mean and standard deviation for firmness and TSS predicted using the calibration and validation sets (N_Calibration_ = 162 samples; N_Validation_ = 48 samples). Structured selection using only spectral information treatment algorithms proved adequate, since the calibration and validation sets displayed similar values for mean, range and standard deviation for all the parameters studied. The prediction performance of PLS, SVM and BPNN models for predicting firmness and TSS content of strawberry fruit are shown in Fig. 3 and Table 2. The performance of these models was evaluated by correlation coefficient (*r*), SEC and SEP. The number of latent factors for PLS model of firmness and TSS was determined as five through comparison of the value of predicted residual error sum of squares (PRESS). Regarding SVM model, optimization of the meta-parameters *C* (regularization parameter) and *G* (RBF kernel parameter) is the key step in SVM as their combined values determine the boundary complexity and the prediction performance. In order to obtain good prediction performance, some parameters in SVM have to be chosen carefully. The best *C* and *G* were found to be 11.31 and 0.06 for firmness prediction, and 2 and 0.06 for TSS content prediction, respectively. For BPNN model, the optimal parameters in modeling process were set as follows after the adjustments of parameters. The number of hidden nodes, the goal error and iteration times were set as 20, 1×10^−8^ and 1,000, respectively.

**Table 1:** Range, mean and standard deviation (SD) for the parameters studied in calibration (N_Calibration_ = 162) and validation (N_Validation_ = 48) sets.

Parameter	Set	Range	Mean	SD
Firmness (N)	Calibration	0.86−7.48	2.65	1.60
	Validation	0.89−6.37	2.28	1.31
TSS (°Bx)	Calibration	5.0−11.4	7.89	1.29
	Validation	6.3−10.6	7.79	0.93

**Table 2:** Performance of PLS, SVM and BPNN models for predicting firmness and TSS.

			Calibration		Prediction	
Parameter	Method	Spectra	SEC	*r*	SEP	*r*
Firmness(N)	PLS	VIS	0.518	0.91	0.451	0.93
		NIR	0.859	0.81	0.943	0.67
		Whole	0.504	0.91	0.421	0.94
	SVM	VIS	0.149	0.94	0.674	0.76
		NIR	1.523	0.78	0.794	0.72
		Whole	0.112	0.94	0.532	0.78
	BPNN	VIS	0.599	0.90	0.436	0.93
		NIR	0.677	0.87	0.713	0.87
		Whole	0.424	0.93	0.375	0.94
TSS (°Bx)	PLS	VIS	0.776	0.64	0.638	0.73
		NIR	0.783	0.55	0.795	0.67
		Whole	0.712	0.81	0.575	0.76
	SVM	VIS	0.756	0.67	0.784	0.60
		NIR	2.780	0.48	0.855	0.42
		Whole	0.361	0.92	0.775	0.62
	BPNN	VIS	0.721	0.59	0.656	0.78
		NIR	0.753	0.58	0.705	0.74
		Whole	0.506	0.76	0.573	0.83

### Firmness Prediction

Compared with PLS and SVM models, BPNN model had the best prediction performance in firmness predicting based on the whole spectra with correlation coefficient (*r*) of 0.93 and 0.94 for calibration and prediction sets, respectively. The SEP and bias in prediction set were 0.375 and 0.035, respectively (Table 2). Although the correlation coefficient in PLS model was similar to that in BPNN model, the SEC and SEP were higher than those in BPNN model indicating that the BPNN model can achieve slightly better prediction accuracy than the PLS model. The results in Table 2 also showed that the prediction models were mostly based on the VIS parts of the spectra and if the NIR parts of the spectra were included the models were slightly improved. Similar findings are reported by Nagata et al. [1], who observed SEP of 0.364 and correlation coefficient (*r*) of 0.784 for the firmness prediction in technically ripe strawberry using hyperspectral imaging in the visible range. Similarly, Tallada et al. [10] conducted a NIR hyperspectral imaging investigation for firmness estimation in strawberry fruit and found that the three-wavelength model had correlation coefficient (*r*) of 0.786 and SEP of 0.350 MPa in 50% to full-ripe group. Furthermore, notice that there was a markedly high variability of firmness values for unripe strawberry fruit and less with the ripe and overripe fruit. As the strawberry fruit mature, aside from increasingly becoming soft, the firmness approaches to a relatively uniform common value.

### Total Soluble Solids Content Prediction

For TSS content prediction, the best prediction performance was also obtained using BPNN model with the correlation coefficient (*r*) of 0.76 and 0.83 for calibration and prediction sets, respectively. The SEP and bias in prediction set were 0.573 and 0.056, respectively (Table 2). Similar to firmness prediction, the VIS parts of the spectra were more important than NIR parts of the spectra and the best models for TSS prediction were attained using the combined VIS and NIR spectra. Our results are consistent with the findings of ElMasry et al. [11], who found correlation coefficient (*r*) of 0.80, SEC of 0.233 and SEP of 0.184 for the prediction of TSS in strawberry fruit using hyperspectral imaging. However, Nagata et al. [1] showed that the TSS calibration models require individual maturity level analysis for more reliable predictions in strawberry using hyperspectral imaging. Therefore, in the present study, an improvement was observed for firmness and TSS prediction in strawberry fruit with the use of multispectral imaging technique and BPNN model compared with previous reports [1], [10], [11]. The improvement was observed for TSS prediction is probably attributed to the analysis approach and the large number of training samples for model development.

### Classification of Fruit Ripeness Stage

**Figure 4:**
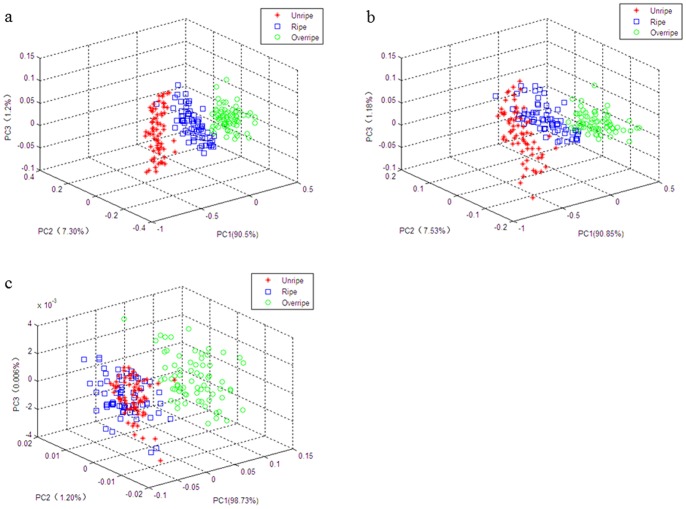
3D principal component score plot for strawberry fruit. Three dimensional score plot of the first three principal components for unripe, ripe and overripe strawberry fruit with the whole wavelength data (a), visual wavelengths data (b), and NIR wavelengths data (c).

Although PCA itself cannot be used as a classification tool, this behavior may indicate the data trend in visualizing dimension spaces. For visualizing the data trends and the discriminating efficiency of the multispectral imaging preprocess, a scatter plot of samples using the first three principal components (PCs) issued from PCA of the data matrix was obtained which are showed in Fig. 4. As can be seen, there is a neat separation of the three ripeness stages of strawberry fruit in the three-dimensional PCA space with the whole wavelengths data. In addition, Fig. 4 also shows the separation was clearer when using the VIS parts of the spectra than that of using the NIR parts of the spectra and if the NIR parts of the spectra were included the separation was slightly improved.

For PCA-BPNN model, the number of hidden nodes, the goal error and iteration times were set as 20, 1×10^−8^ and 1,000, respectively. Table 3 shows the classification results of three ripeness stages of strawberry fruit in the prediction set using SVM and PCA-BPNN models, respectively. The results showed that the classification accuracy of BPNN model was achieved 93.33%.

**Table 3:** Classification accuracies of fruit ripeness stage using SVM and PCA-BPNN models.

Sample	Accuracy of SVM model (%)	Accuracy of PCA-BPNN model (%)
Unripe	100	100
Ripe	100	90
Overripe	100	90
Total	100	93.33

For SVM model, the best *C* and *G* were found to be 0.25 and 2, respectively. The results showed that high classification accuracy of 100% was achieved using SVM model, which was higher than that of using PCA-BPNN model (Table 3). The classification accuracy was much higher than that of ElMasry’s study (89.61%) [11]. This increased accuracy is very important for classification of fruit ripeness stage in practical applications. Furthermore, compared to hyperspectral imaging system [11], the multispectral imaging system can shorten the image acquisition and processing time, enabling real-time automated quality monitoring systems.

### Conclusion

In this paper a multispectral imaging system and different analysis approaches were investigated to examine the possibility of using multispectral imaging to assess the quality attributes and ripeness stage in strawberry fruit. Compared with PLS and SVM models, BPNN model for the firmness (*r* = 0.94, SEP = 0.375 and bias = 0.035) and TSS content (*r* = 0.83, SEP = 0.573 and bias = 0.056) demonstrated good prediction performance. High classification accuracy of 100% for correctly identifying ripeness stage of strawberry fruit was achieved using SVM model, which was higher than that of using PCA-BPNN model. Moreover, all these models were mostly based on the VIS parts of the spectra and if the NIR parts of the spectra were included the models were slightly improved. In conclusion, the present study has shown that multispectral imaging is a promising nondestructive method for rapid analysis of the quality attributes and ripeness stage of strawberry fruit.
